# Cylindrical vector beam multiplexing holography employing spin-decoupled phase modulation metasurface

**DOI:** 10.1515/nanoph-2023-0731

**Published:** 2024-02-07

**Authors:** Zhiqiang Xie, Zeming Liang, Haisheng Wu, Qingji Zeng, Zhiwei Guan, Aofan Long, Pin Zhong, Junmin Liu, Huapeng Ye, Dianyuan Fan, Shuqing Chen

**Affiliations:** Institute of Microscale Optoelectronics, Shenzhen University, Shenzhen 518060, China; College of New Materials and New Energies, Shenzhen Technology University, Shenzhen 518118, China; Guangdong Provincial Key Laboratory of Optical Information Materials and Technology and Institute of Electronic Paper Displays, South China Academy of Advanced Optoelectronics, South China Normal University, Guangzhou 510006, China

**Keywords:** multiplexing holography, cylindrical vector beams, spin-decoupled phase modulation, metasurface

## Abstract

Cylindrical vector beams (CVBs) hold considerable promise as high-capacity information carriers for multiplexing holography due to their mode orthogonality. In CVB holography, phase holograms are encoded onto the wave-front of CVBs with different mode orders while preserving their independence during reconstruction. However, a major challenge lies in the limited ability to manipulate the spatial phase and polarization distribution of CVBs independently. To address this challenge, we propose a spin-decoupled phase modulation strategy by leveraging the propagation and geometric phase of composite phase metasurfaces. By exploiting the polarized Poincaré sphere, we show that CVBs can be decomposed into two circularly polarized components with orthogonal polarization states and conjugate phase distributions. This decomposition enables independent control of the phase and polarization distributions of CVBs by modulating the initial phase and phase difference of these two components. Consequently, two holograms with discrete spatial frequency distributions that carry opposite helical phases are encoded to modulate the wave-front of CVBs by the metasurface consisting of Si nanopillars. This allows for us to achieve successful four-channel CVB multiplexing holography. Benefiting from the non-dispersive nature of geometric phase, this metasurface exhibits a broad operating band spanning the entire visible light spectrum (443 nm–633 nm). These suggest that our proposed method offers comprehensive control over the spatial phase and polarization of CVBs, thereby holding significant potential for advancing their application in holography.

## 1 Introduction

Cylindrical vector beams (CVBs) [1], [2], [3], [4] with spatially varying polarization distributions offer a promising approach for achieving high-capacity holography through mode multiplexing. In multiplexing holography, distinct phase holograms are encoded onto the wave-front of light beams, and holographic images are reconstructed by leveraging the orthogonality of the multiplexing dimensions [5], [6], [7], [8], [9], [10]. For instance, wavelength multiplexing holography can be realized using a three-dimensional nonlinear photonic crystal, which exploits the quasi-phase-matching of nonlinear Ewald construction in reciprocal space to encode multiple holograms at different wavelengths [11]. By satisfying the corresponding quasi-phase-matching condition, holographic images can be selectively reconstructed at the second-harmonic wave. Sophisticated device structures and algorithmic optimization of holograms can also be employed to leverage physical dimensions such as polarization [12], [13], incident angle [14], and transmission direction [15] for multiplexing holography. However, the orthogonality of CVB modes arises from their distinct spatial polarization distributions. The fundamental requirement of CVB holography is to encode holograms onto the wave-front of CVBs while preserving the integrity of their spatial polarization structures throughout the reconstruction process. In recent developments, vortex beams carrying orbital angular momentum (OAM) modes has been utilized for holography [16], [17], [18], [19]. By increasing the spatial frequency shift in k-space, the preservation of OAM modes in reconstructed holographic images can be achieved, providing valuable inspiration for the realization of CVB holography. However, unlike the spatial phase distribution of OAM modes, which can be modulated using phase modulation device such as diffractive optical elements [20], [21], spiral phase plates [22], and spatial light modulators (SLMs) [23], independently manipulating the phase and polarization distributions of CVBs has remained a challenge due to the limited availability of phase-only spatial modulation devices.

Modulating CVB modes typically requires devices with spatially-varying polarization properties, such as axially birefringent components [24], [25], subwavelength gratings [26] and spatially varying retarders [27]. For example, a calcite crystal aligned along the propagation direction in a laser cavity has been used to generate CVB modes [28]. Similarly, devices with spatially-varying polarization properties can be employed to convert homogeneous polarization distributed light beams into CVBs [29], [30]. However, these modulation methods for CVB modes have primarily focused on achieving arbitrary modulation of the polarization distribution while often neglecting the spatial phase modulation. Metasurfaces are planar artificial materials consisting of subwavelength nanopillars capable of locally manipulating the phase, amplitude, and polarization states of light [31], [32], [33]. The subwavelength scale of these unit cells enables metasurfaces to provide exceptional spatial resolution, a large information capacity, and a wide field of view, making them highly suitable for holographic imaging applications [34], [35], [36], [37], [38]. By leveraging the polarization Poincaré sphere theory, arbitrary polarization states can be decomposed into left circularly polarization (LCP) and right circularly polarization (RCP) components. Independent phase and polarization control of light beams can be achieved by controlling the initial phase and phase difference of these two components. Therefore, we propose a spin-decoupled phase modulation strategy by exploiting the propagation and geometric phase of composite phase metasurfaces. The sizes and orientations of nanopillars are tailored to modulate the initial phase and phase difference of the LCP and RCP components of CVBs. Additionally, to prevent the overlap of CVBs in adjacent pixels of the reconstructed holographic image, a two-dimensional (2D) Dirac comb function with a sampling period (p) is utilized to discretize the holographic images, and the CVB multiplexing holography is achieved by superposing holograms associated with distinct CVB modes. Compare to traditional vectorial holography, which reconstructed holographic images of continuous distributed but with spatially-variant polarizations by using Diatomic metasurface [39], [40]. Our method using the CVB as the carriers of holographic image, which infinite holographic images can be multiplexed in one meta-hologram in theory.

To verify its feasibility, we utilized subwavelength Si nanopillars on a fused silica to fabricate designed meta-holograms. Two holograms with conjugated helical phases were loaded onto the two spin components, effectively encoding the holography information onto the incident light beam. We demonstrate that specific polarized orders of CVBs were carried by the incident light beam and accurately replicated to each pixel of the reconstructed holographic image. Building upon this foundation, we further superposed four holograms with different CVB modes (*m* = −2, −1, 1, 2) to achieve four-channel CVB multiplexing holography. By illuminating with different CVB modes spanning from −2 to 2, holographic images corresponding to each mode were successfully reconstructed. Notably, the CVB meta-hologram operates across the entire visible light spectrum (range from 443 nm to 633 nm) benefiting from the non-dispersibility of geometric phase. These indicate that this approach offers a versatile and precise method for effectively controlling vector fields, as it enables the manipulation of both the polarization and phase distribution of CVBs. Furthermore, the integration of metasurfaces enhances the potential applications of this approach in various fields, including vectorial holography, data storage, optical encryption, and optical communications.

## 2 Principles and methods

According to Fourier transform holography, the spatial frequency distribution of a hologram corresponds to the electric field distribution in the image plane. The relationship between the electric field distribution in the image plane and the incident electric field can be express as:

(1) $$\begin{array}{ll} E_{\mathrm{image}} & =f\;ft\left(E_{H}\left(k_{x},k_{y}\right)\cdot E_{in}\right)\\ & =f\;ft\left(E_{H}\left(k_{x},k_{y}\right)\right)*f\;ft\left(E_{in}\right), \end{array}$$

where *E*_*image*_ is the electric field distribution in the image plane, *E*_*H*_(*k*_*x*_, *k*_*y*_) is the constituent spatial-frequency components comprising the hologram, *E*_*in*_ is the incident electric field, and (*k*_*x*_, *k*_*y*_) represents the orthogonal coordinates in the hologram plane. From Equation (1), it can be observed that the reconstructed electric field distribution in the image plane is obtained through the convolution between the holographic image and the Fourier transform of the incident beam. However, in the case of conventional digital holograms, which have a quasi-continuous spatial frequency distribution, the light spot of the CVB is larger than the size of a single pixel. As a result, the polarization and light intensity distribution of the CVB will be destroyed due to the overlapping and interference of adjacent CVBs (for more detailed information, please refer to Supplementary Note 1). In order to avoid spatial overlap of the polarization state of adjacent CVBs, a linear spatial frequency shift can be introduced to the incident beam (*k*_in_). This shift helps preserve the complete polarization distribution in each pixel of a reconstructed holographic image. Therefore, the conventional digital hologram with a quasi-continuous spatial frequency distribution needs to be discretized.

**Figure 1:**
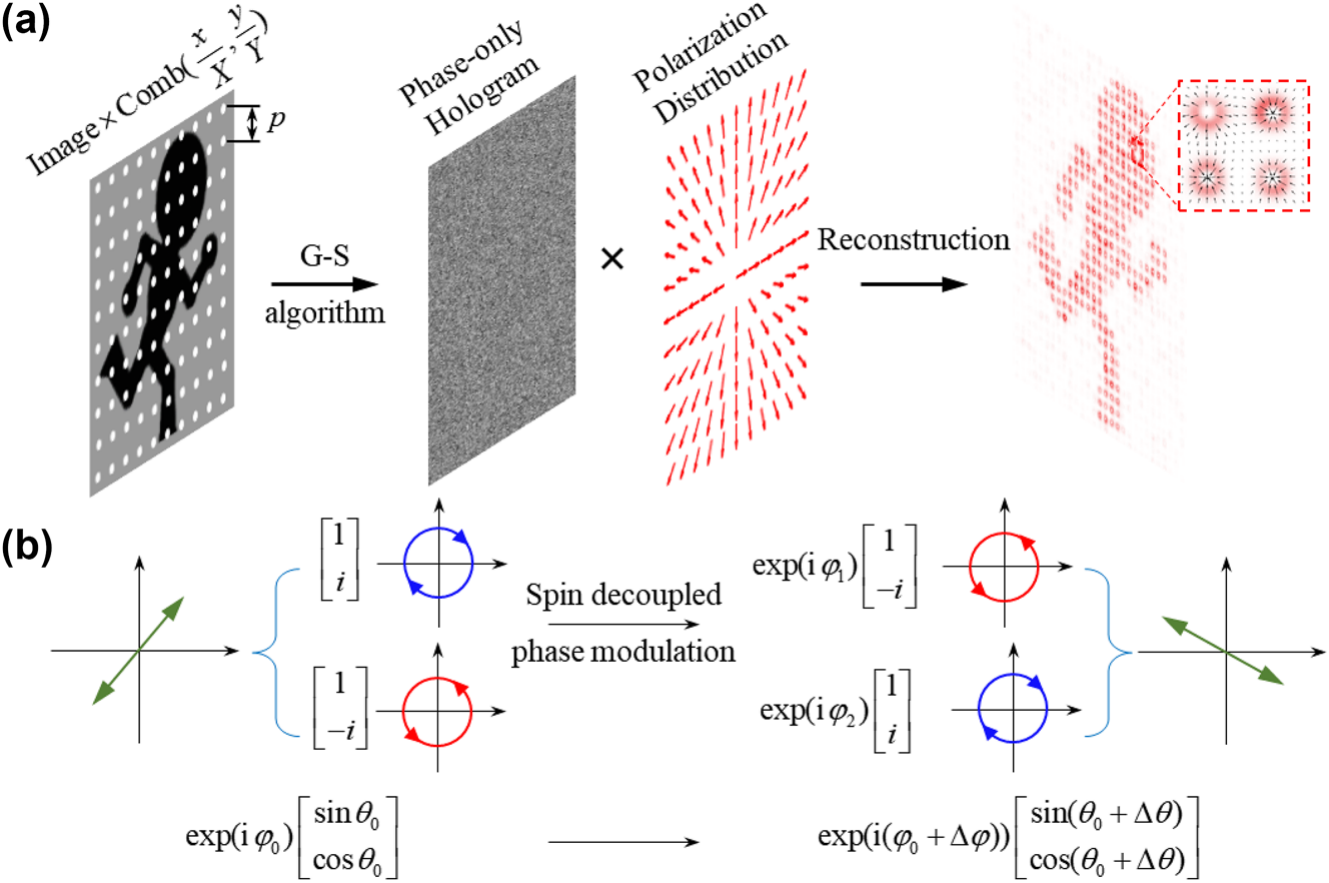
Principle of CVB holography based on the independent phase and polarization modulation. (a) Schematic illustration of designing and reconstruction of CVB holography, which contains a digital phase hologram and a 2D vector field of CVB; (b) schematic of independent phase and polarization modulation of light beams using spin-decoupled phase modulation.

The physical mechanism of the CVB holography we designed is demonstrated and illustrated in Figure 1(a). To maintain the polarization distribution of the incident CVB on adjacent pixels in the holographic image, we spatially sampled the object image using a 2D Dirac comb function, where *p* is the period constants. As a result, the reconstructed electric field distribution in the image plane can be expressed as a convolution between the holographic image and the CVB (see in Supplementary Note 2). Consequently, the CVB hologram can be obtained by performing a Fourier transform of the product between hologram of the target image and the polarization distribution of the CVB. In this case, the modulation of the light field required to achieve CVB holography can be described by:

(2) $$E=\exp\left(i\Phi \right)\left[\begin{array}{c} \sin m\theta \\ \cos m\theta \end{array}\right]$$

where Φ represents the phase-only hologram of discrete target image (it can be attained by utilizing the G-S iterative algorithm), $\left[\begin{array}{c} \sin m\theta \\ \cos m\theta \end{array}\right]$ represents the polarization distribution of CVB modes, and *m* is the polarization order. Finally, the holographic image composed of CVBs can be reconstructed using the designed CVB hologram. It is important to note that the period constants *p* is associated with the polarization order *m*, as the beam waist of the CVB in the image plan is proportional to the polarization order (see Supplementary Note 3). additionally, CVB holography can be considered as a form of Fourier holography, and thus the period constants *p* is also influenced by the effective numerical aperture (*NA* = sin(*atan*(*R*/*f*))) and the wave number *k* of the incident beam.

From the Eq. (2), it can be inferred that the key to realizing CVB holography is the independent control of the phase and polarization of the incident CVB beam. Base on the Poincaré sphere principle of polarization, LCP and RCP components serve as a pair of polarization vectors. Any arbitrary polarization state can be decomposed into LCP and RCP components with different phase differences. The Jones matrix can be expressed as follows:

(3) $$\begin{array}{ll} \exp\left(i\varphi_{0}\right)\left[\begin{array}{c} \cos\left(\theta \right)\\ \sin\left(\theta \right) \end{array}\right] & =\frac{1}{2}\exp\left(i\theta_{0}\right)\left[\begin{array}{c} \exp\left(i\theta \right)+\exp\left(-i\theta \right)\\ i\exp\left(-i\theta \right)-i\exp\left(+i\theta \right) \end{array}\right]\\ & =\frac{1}{2}\exp\left(i\theta_{0}\right)\exp\left(-i\theta \right)\left[\begin{array}{c} 1\\ i \end{array}\right]\\ & \;+\frac{1}{2}\exp\left(i\theta_{0}\right)\exp\left(i\theta \right)\left[\begin{array}{c} 1\\ -i \end{array}\right], \end{array}$$

where exp(*iφ*_0_) represents the initial phase, and $\left[\begin{array}{c} \sin\theta \\ \cos\theta \end{array}\right]$ represents the polarization distribution with the orientation angle *θ*. In this case, as shown in Figure 1(b), the spin decoupled phase modulation method is used to encode exp(*iφ*_1_) and exp(*iφ*_2_) to the LCP and RCP components, respectively. Thus, the output optical filed can be expressed by $\exp\left(i\left(\varphi_{0}+\Delta \varphi \right)\right)\left[\begin{array}{c} \sin\left(\theta_{0}+\Delta \theta \right)\\ \cos\left(\theta_{0}+\Delta \theta \right) \end{array}\right]$ , where $\Delta \varphi =\frac{\varphi_{1}+\varphi_{2}}{2}$ , $\Delta \theta =\frac{\varphi_{2}-\varphi_{1}}{2}$ . It indicates that both phase and polarization are manipulated simultaneously. This enables spatially independent modulation of both polarization and wavefront by independently controlling the phase difference and initial phase of the two spin eigenstates.

**Figure 2:**
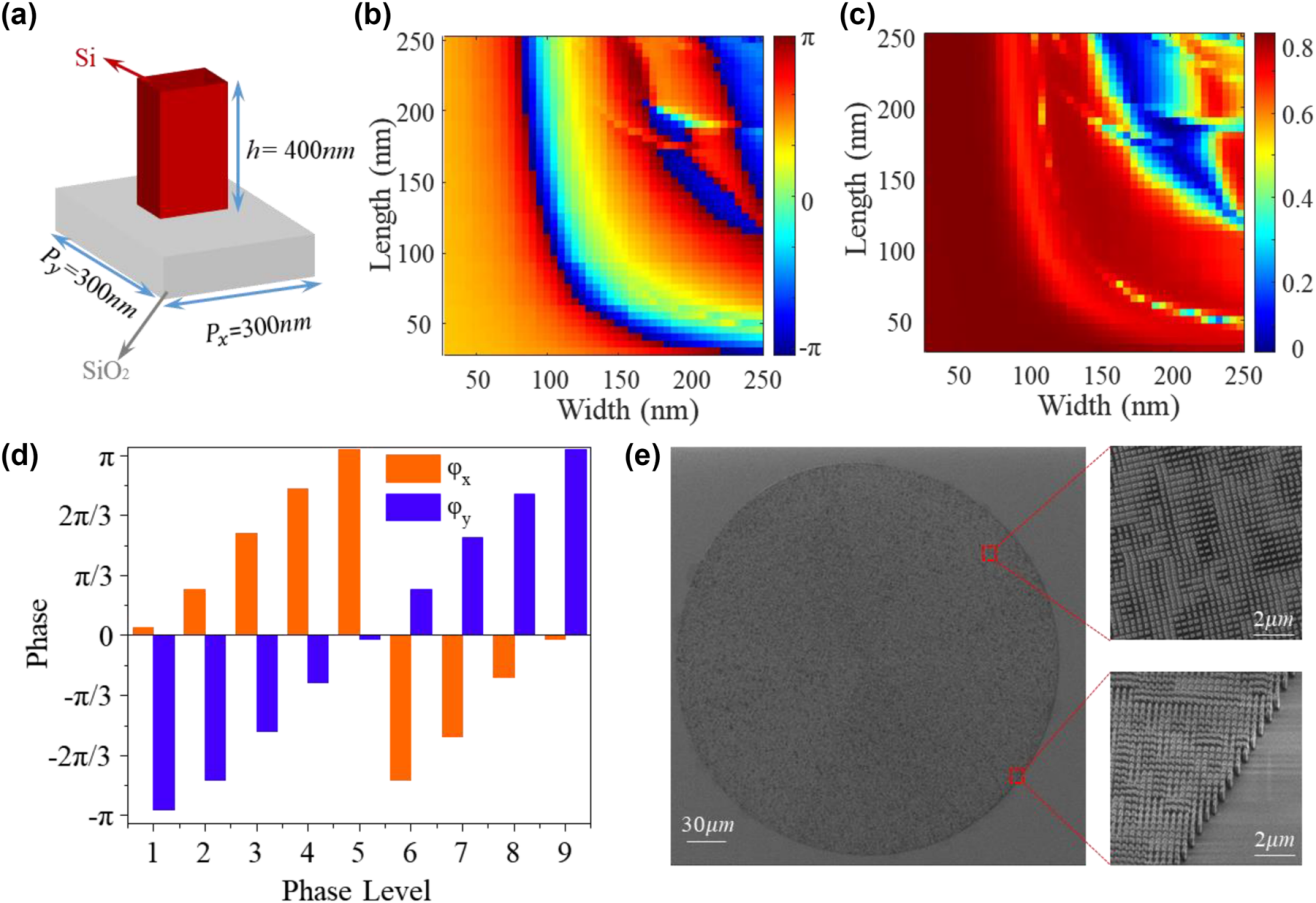
Design and fabrication of the CVB-selective meta-hologram. (a) Schematic illustration of a silicon nanopillar positioned on a glass substrate; (b) and (c) numerical characterization of the phase and transmission coefficients of *x*-polarized light beam transmitted from silicon nanopillar with different width and lengths. (d) Calculated phase shifts *φ*_*x*_ and *φ*_*y*_, for different phase levels at the wavelength of 633 nm. (e) SEM image of the fabricated CVB meta-hologram.

Here we realize spin-decoupled phase modulation by combining propagation with geometric phase metasurface. This approach leverages the independent control of the size and azimuth of each nanopillars, allowing for spatially independent phase modulation of both LCP and RCP components (for more details, please refer to Supplementary Note S4). To verify the feasibility of the aforementioned methods, a Si–SiO_2_ based metasurface was designed. Figure 2(a) illustrates the metasurface, which consists of a silicon nanopillar with a height of 400 nm positioned on a SiO_2_ substrate. The lattice of the metasurface is 300 × 300 nm^2^, which the lattice remains non-diffractive and satisfies the Nyquist sampling criterion. As show in Figure 2(b) and (c), the phase retardation and amplitude of *x*-polarized light beam transmitted from a Si nanopillar were numerically simulated with periodic boundary conditions as a function of nanopillar width and length. These results were obtained using finite difference time domain (FDTD) method (see Supplementary Note S8 for more details). The Si–SiO_2_ metasurface demonstrate both high transmission efficiency and complete phase modulation ([0, 2π]), which are essential for achieving high diffraction efficiency in meta-holograms. It is worth noting that to achieve the metasurface enables the transformation from LCP to HCP, the unit structure should also satisfy the half-wave plate effect ( $\left|\varphi_{x}-\varphi_{y}\right|=\pi$ , where *φ*_*x*_ and *φ*_*y*_ are the propagation phase retardation on the *x*- and *y*-direction linear polarization, respectively). In the experiment, we selected nine different sized nanopillars to design and fabricate a nine-level CVB based meta-hologram with length and width of (95 nm, 135 nm), (95 nm, 160 nm), (150 nm, 60 nm), (130 nm, 85 nm), (135 nm, 95 nm), (155 nm, 95 nm), (60 nm, 150 nm), (85 nm, 130 nm), (95 nm, 130 nm). The phase retardation of *φ*_*x*_ and *φ*_*y*_ scattered from Si nanopillars at a wavelength of 633 nm are depicted in Figure 2(c). It is evident that the phases of both *φ*_*x*_ and *φ*_*y*_ are uniformly distributed between 0 to 2π and satisfying the half-wave condition as well. This characteristic is crucial for the design and fabrication of nine-level CVB meta-holograms. Based on these simulated parameters and desired phase modulation of LCP and RCP, the layout of entire metasurface can obtained. To verify the feasibility of metasurface-based CVB holography, we fabricated the designed Si–SiO_2_ dielectric metasurfaces using electron beam lithography and plasma etching techniques. The physical size of the fabricated metasurface is a circular with diameter of 300 µm. Figure 2(d) shows the scanning electron microscopy (SEM) images of the fabricated CVB meta-hologram. The top-view and oblique-view SEM images of enlarged areas within the meta-hologram are presented on the right side. More details about the fabrication process can be found in Supplementary Note S7.

**Figure 3:**
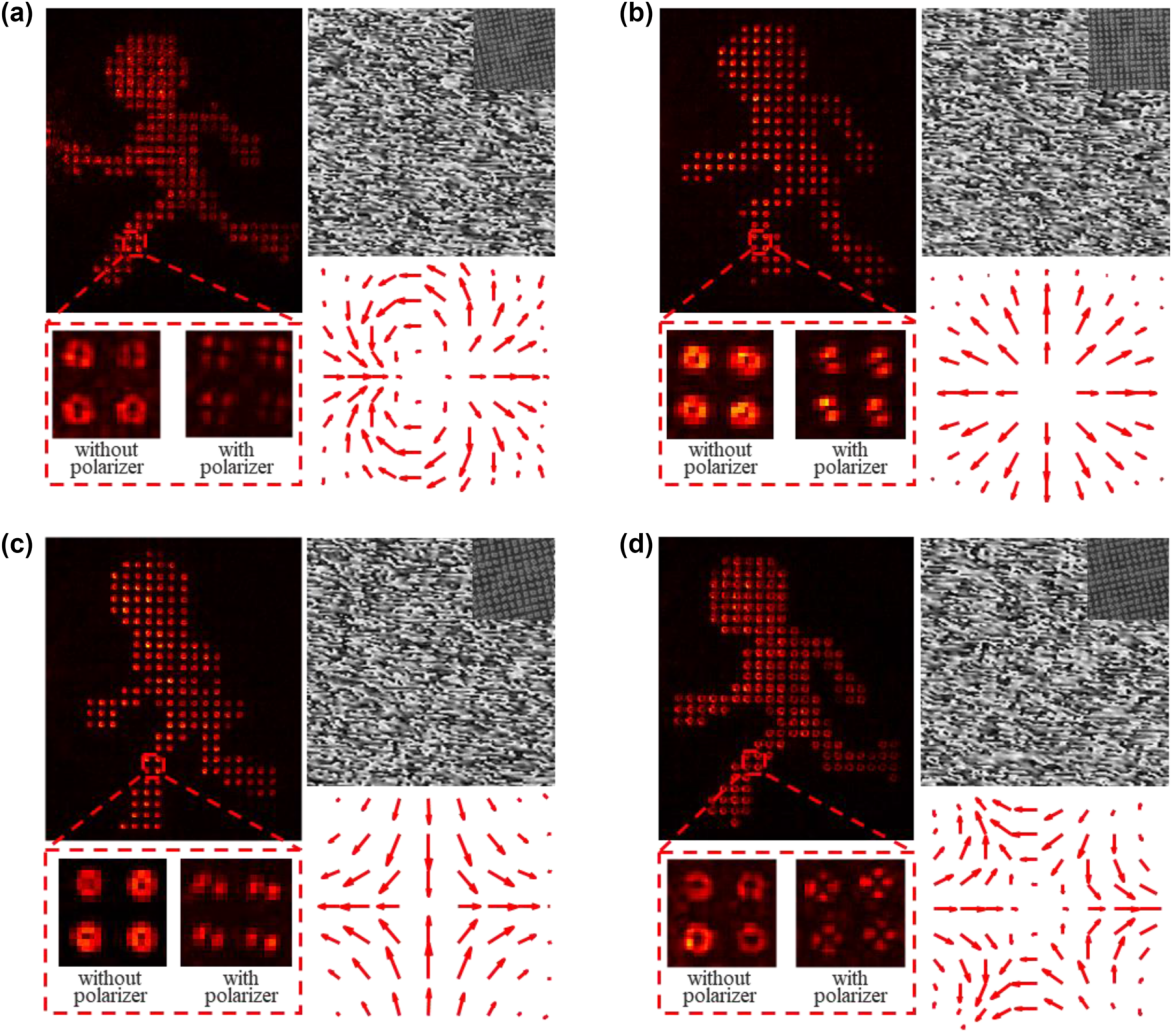
Experimental characterization involved reconstructing holographic images carrying CVBs (*m* = −2, −1, 1, and 2) from various CVB-dependent meta-holograms using incident beams with linearly polarization. (a)–(d) Reconstructed holographic image and corresponding SEM images of the CVB meta-holograms.

To demonstrate the effectiveness of the proposed CVB holography scheme, we designed and fabricated four CVB meta-holograms that encode four running states of a stick figure into CVB modes with different orders (*m* = −2, −1, 1, 2). The optical performance of these meta-holograms was characterized using an experimental setup described in Supplementary Note S6. Figure 3(a)–(d) depict the reconstructed holographic images obtained when a linearly polarized Gaussian beam is incident normally on the meta-holograms. By manipulating the phase and polarization of the incident beam using the metasurface, CVB modes with specific polarized orders are imparted onto the incident light beam and replicated in each pixel of the reconstructed holographic image. The intensity distribution of CVBs in the pixels is shown within the red dashed box. Based on the polarization detection results, the polarization of the CVBs in each holographic image correspond to *m* = −2, −1, 1, 2, respectively. We further calculated the polarization distribution of CVB mode in a single pixel using the method of Stokes parameters [41], [42], more experimental results can be seen in the Supplementary Note S11. The theoretical phase hologram and polarization distribution are shown on the right side of Figure 3(a)–(d), along with the SEM image of the central area of the metasurface. From the arrangement of the metasurface nanopillars, it is evident that the CVB meta-hologram exhibits not only changes in size but also changes in rotation angle. The rotation angle corresponds to half of the polarization state of the encoded CVB. These results are in good agreement with our expectations and validate the feasibility of our designed CVB holographic scheme. Furthermore, based on the conservation of modes and the spatial and intensity differences between a fundamental spatial mode and high-order CVB modes, the CVB meta-holograms possess strong selectivity in imaging (for more detail, refer to Supplementary Note S9). It is worth noting that the powerful selectivity imaging of CVB meta-holograms is a fundamental requirement for achieving CVB multiplexing holography.

**Figure 4:**
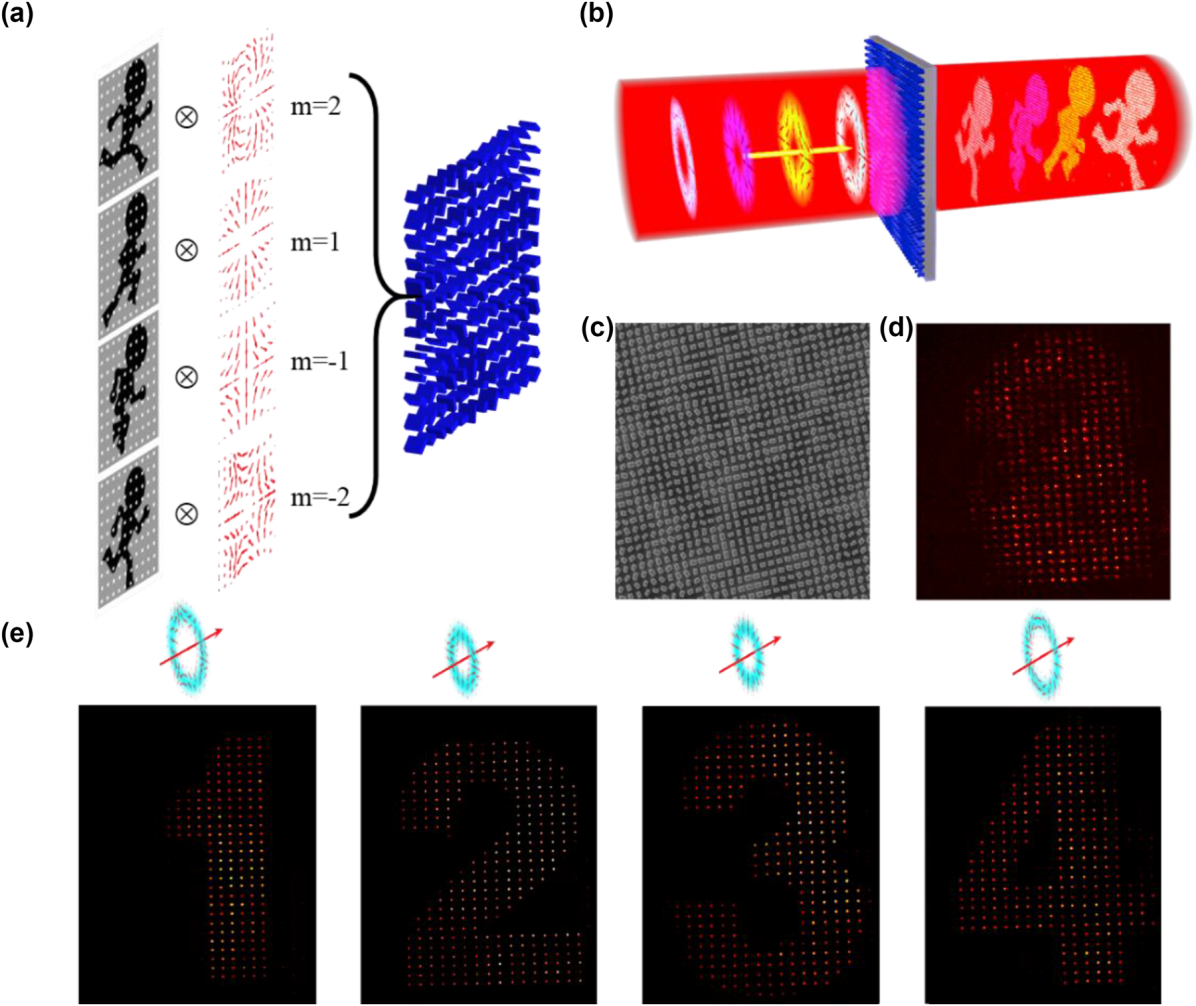
Design and experimental demonstration of the four-channel CVB multiplexed meta-hologram. (a) Design approach of an CVB-multiplexing hologram; (b) different holographic images can be obtained with different CVB mode incident the meta-hologram. (c) SEM images of the center areas of meta-hologram. (e) The reconstruction of four distinctive CVB-dependent holographic images through incident CVBs with polarization orders of *l* = −2, −1, 1, 2, respectively.

After correlating the CVB modes with the designed images, we can extend the capability of holography by achieving optical multiplexing through the use of orthogonal CVB modes. The multiplexing method is schematically illustrated in Figure 4(a), where four target images are discretized using a 2D Dirac function and added via different polarization distributions of CVB modes (*m* = −2, −1, 1, 2). In this scenario, the modulation of the light field required to achieve CVB multiplexing holography can be described by:

(4) $$E=\overset{n}{\underset{k=1}{\sum }}\exp\left(i\varphi_{k}\right)\left[\begin{array}{c} \sin m_{k}\theta \\ \cos m_{k}\theta \end{array}\right].$$

Hence, the phase modulate of two spin components should be:

(5) $$\varphi_{\mathrm{LCP}}\left(x,y\right)=\overset{n}{\underset{k=1}{\sum }}\exp\left(i\varphi_{k}\right)\exp\left(im_{k}\theta \right)$$

(6) $$\varphi_{\mathrm{RCP}}\left(x,y\right)=\overset{n}{\underset{k=1}{\sum }}\exp\left(i\varphi_{k}\right)\exp\left(-im_{k}\theta \right)$$

In this case, the phase and polarization modulate of meta-hologram can be separately expressed as:

(7) $$\varphi \left(x,y\right)=\frac{\varphi_{\mathrm{LCP}}\left(x,y\right)+\varphi_{\mathrm{RCP}}\left(x,y\right)}{2},$$

(8) $$\vartheta \left(x,y\right)=\frac{\varphi_{\mathrm{LCP}}\left(x,y\right)-\varphi_{\mathrm{RCP}}\left(x,y\right)}{2},$$

where the *φ*(*x*, *y*) represent the phase modulate, *ϑ*(*x*, *y*) represent the polarization modulate. The size and rotation angle of the metasurface can be calculated based on the phase and polarization modulation requirements. To illustrate this, we will consider the example of compatible CVB meta-hologram multiplexing. In this case, four Arabic numerals (“1”, “2”, “3”, and “4”) were sampled in the spatial frequency domain. For the LCP and RCP components, their holograms were encoded with spiral phase plates with topological charges of *l* = −2, −1, 1, 2 (*l* = 2, 1, −1, and −2), respectively. To superpose the four holograms of LCP and RCP, a spin-decoupled phase modulation is used to integrate them into one CVB meta-hologram. Consequently, when an incident beam with a linearly polarized Gaussian profile interacts with the CVB-multiplexing meta-hologram, a complex interference pattern can be reconstructed, as demonstrated in Figure 4(d). It cannot find any holographic information. Owing to the CVB mode conservation, only a given CVB mode with an inverse polarization order can be converted to Gaussian mode with stronger intensity distribution in each pixel of the holographic image. In this case, CVBs with polarization orders of *m* = −2, −1, 1, and 2 can selectively and unambiguously reconstruct four distinctive CVB-dependent holographic images from the CVB-multiplexing meta-hologram, as shown in Figure 4(e). To reduce the crosstalk between each holographic image channels, the aperture array can be used to filter out fundamental spatial mode in each pixel of the holographic image (more detail can be seen in Supplementary Note S9). In this case, we further calculated the signal-to-noise ratio (SNR) of each channel, there are 11.5 dB, 14.8 dB, 11.3 dB, and 9.16 dB, respectively (more detail can be seen in Supplementary Note S10).

## 3 Discussion

**Figure 5:**
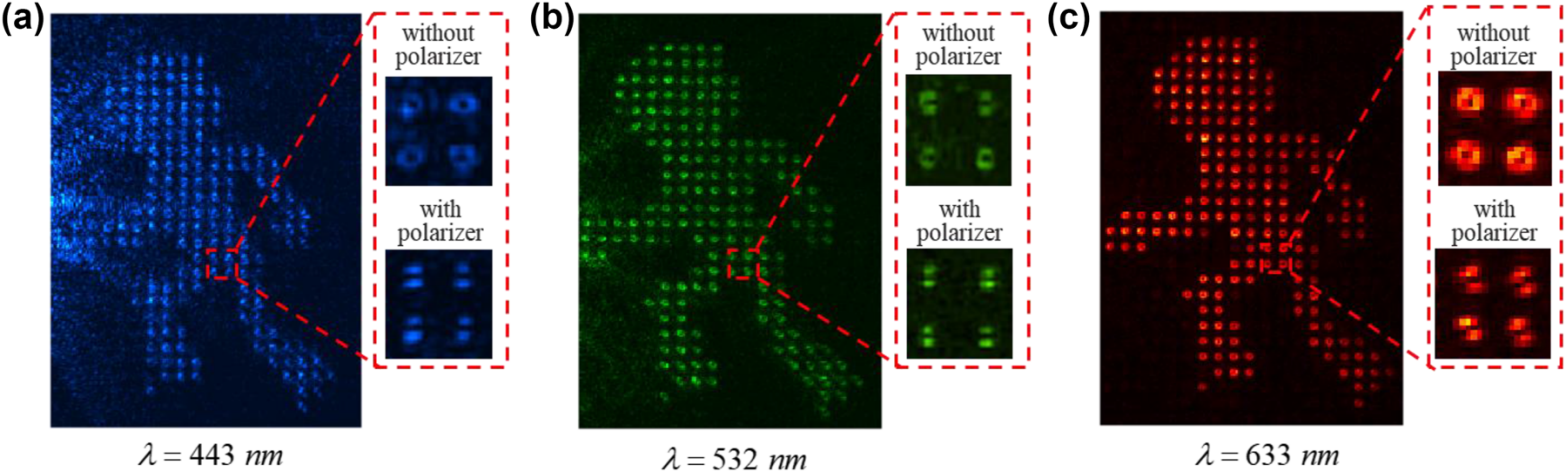
Reconstructed holographic image with different wavelength (443 nm, 532 nm, 633 nm).

Our demonstration represents a significant advancement in metasurface holography by harnessing the previously inaccessible CVB mode as an independent information carrier. We achieve this by discretizing the target image using a 2D comb function and associating it with the CVB mode. Furthermore, we are achieving multiplexing of multiple holograms by leveraging the orthogonality of the CVB modes. The key to our approach lies in the independent modulation of the phase and polarization of CVB modes, which is achieved by combining propagation and geometric phase metasurface. While theoretically, the multiplexing channels of CVB holography are infinite our designed CVB holography remain essentially a phase-based hologram. This limitation arises from the lack of exact convolution between a complex-amplitude image channel and the CVB wavefront, resulting in increased crosstalk between channels and limiting the amount of multiplexing. To address this, the interference effect among multi meta-atoms has been demonstrated to achieve spin-decoupled complex-amplitude modulation [43]. This enables independent modulation of the complex-amplitude phase and polarization by manipulating the initial phase and phase difference between the LCP and RCP components. Additionally, the high refractive index of Si allows us to have full control over the phase of the CVB wavefront, with phase changes spanning from 0 to 2π, and the real part of refractive index equal to 0.019, which indicated that it has high transmittance. In addition, as a common semiconductor material, Si has a relatively fledged etching process, which can be prepared in large quantities to meet practical needs. Furthermore, we have tested the wavelength response range of the CVB meta-holograms, as shown in Figure 5. The results demonstrate that the wavelength response range of the CVB meta-holograms covers the entire visible light band, ranging from 443 nm to 633 nm. However, the initial phase modulation is caused by the propagation phase, which is wavelength sensitive. And in this work, the working band is set as 633 nm while designing the CVB holography metasurfaces. Hence, when the wavelength of 443 nm light beam as the incident, the efficiency will decrease and shows the poor performance.

The synthesis of the vector mode using two OAM modes enables the demultiplexing of CVB holography when the OAM mode is incident, following the mode conservation law. This demonstrates the compatibility between CVB and OAM in our proposed CVB holographic solution. However, it also highlights the potential insecurity issues of this design solution. To address this concern, one possible approach to encrypt CVB holographic information is to divide the target image into two parts and assign each part to the left and right spin components of the CVB pattern, respectively. This encryption method can also be extended to cylindrical vector vortex beams or other complex light beams as holography information carriers. By incorporating a mode dynamic modulation scheme [44], [45], dynamic holography can be achieved, allowing for the creation of intricate and visually captivating holographic projections with a wide range of applications. It is worth noting that our phase and polarization independent modulation strategy represents a highly versatile and effective solution for waveform modulation. This strategy offers significant advantages not only in holography but also in other applications such as optical image processing, optical communication, and virtual reality.

## 4 Conclusions

In summary, we have proposed and experimentally demonstrated a method for multiplexing holography using CVBs. This method utilizes a phase and polarization independent modulation technique enabled by a spin-decoupled phase modulation metasurface. By applying the conservation law of CVB modes, we have shown that only a specific CVB mode with inverse polarization order (−*m*) can be used to reconstruct the target holographic image via converting back to a Gaussian mode. This unique property enhances confidentiality during image demultiplexing. Furthermore, our meta-hologram takes advantage of the non-dispersive nature of geometric phase, allowing it to operate across a wide range of wavelength spanning the entire visible light spectrum. These characteristics make our method practical and versatile, with potential applications in various CVB-related fields such as multiplexing holography, optical communication, data storage, and optical encryption.

## Supplementary Material

Supplementary Material Details

## References

[1] Zhan Q. (2009). Cylindrical vector beams: from mathematical concepts to applications. *Adv. Opt. Photonics*.

[2] Chen S. (2021). Cylindrical vector beam multiplexer/demultiplexer using off-axis polarization control. *Light: Sci. Appl.*.

[3] Jia J. (2021). Arbitrary cylindrical vector beam generation enabled by polarization-selective Gouy phase shifter. *Photonics Res.*.

[4] Xian M., Xu Y., Ouyang X., Cao Y., Lan S., Li X. (2020). Segmented cylindrical vector beams for massively-encoded optical data storage. *Sci. Bull.*.

[5] Li X. (2023). Time-sequential color code division multiplexing holographic display with metasurface. *Opto-Electron. Adv.*.

[6] Deng J. (2019). Spatial frequency multiplexed meta-holography and meta-nanoprinting. *ACS Nano*.

[7] Shi Y. (2022). Augmented reality enabled by on‐chip meta‐holography multiplexing. *Laser Photonics Rev.*.

[8] Lin X. (2023). Time-sequential color code division multiplexing holographic display with metasurface. *Opto-Electron. Adv.*.

[9] Chen J., Wang D., Si G., Teo S. L., Wang Q., Lin J. (2023). Planar peristrophic multiplexing metasurfaces. *Opto-Electron. Adv.*.

[10] Jin Z. (2021). Phyllotaxis-inspired nanosieves with multiplexed orbital angular momentum. *eLight*.

[11] Chen P. (2021). Quasi-phase-matching-division multiplexing holography in a three-dimensional nonlinear photonic crystal. *Light: Sci. Appl.*.

[12] Wang Q. (2018). Reflective chiral meta-holography: multiplexing holograms for circularly polarized waves. *Light: Sci. Appl.*.

[13] Barrera J. F., Henao R., Tebaldi M., Torroba R., Bolognini N. (2006). Multiplexing encrypted data by using polarized light. *Opt. Commun.*.

[14] Jang J., Lee G., Sung J., Lee B. (2021). Independent multichannel wavefront modulation for angle multiplexed meta‐holograms. *Adv. Opt. Mater.*.

[15] Shang G. (2021). Transmission–reflection-integrated multiplexed Janus metasurface. *ACS Appl. Electron. Mater.*.

[16] Shi Z., Wan Z., Zhan Z., Liu K., Liu Q., Fu X. (2023). Super-resolution orbital angular momentum holography. *Nat. Commun.*.

[17] Kong L., Sun Y., Zhang F., Zhang J., Zhang X. (2023). High-dimensional entanglement-enabled holography. *Phys. Rev. Lett.*.

[18] Fang X., Ren H., Gu M. (2020). Orbital angular momentum holography for high-security encryption. *Nat. Photonics*.

[19] Li F., Ding H., Nie S., Ma J., Yuan C. (2023). Multiple-image encryption using phase jump gradient factors -based OAM multiplexing holography. *Opt. Lasers Eng.*.

[20] Ruffato G., Massari M., Romanato F. (2019). Multiplication and division of the orbital angular momentum of light with diffractive transformation optics. *Light: Sci. Appl.*.

[21] Hu Z. (2022). Broad-bandwidth micro-diffractive optical elements. *Laser Photonics Rev.*.

[22] Khonina S. N., Podlipnov V. V., Karpeev S. V., Ustinov A. V., Volotovsky S. G., Ganchevskaya S. V. (2020). Spectral control of the orbital angular momentum of a laser beam based on 3D properties of spiral phase plates fabricated for an infrared wavelength. *Opt. Express*.

[23] Zhang N., Xiong B., Zhang X., Yuan X. (2023). High-capacity and multi-dimensional orbital angular momentum multiplexing holography. *Opt. Express*.

[24] Liu S., Qi S., Li Y., Wei B., Zhao J. (2022). Controllable oscillated spin Hall effect of Bessel beam realized by liquid crystal Pancharatnam-Berry phase elements. *Light: Sci. Appl.*.

[25] Lin Z. (2021). Cylindrical vector beam sorter with spin-dependent spiral transformation. *Opt. Lett.*.

[26] Chen W., Han W., Abeysinghe D. C., Nelson R. L., Zhan Q. (2011). Generating cylindrical vector beams with subwavelength concentric metallic gratings fabricated on optical fibers. *J. Opt.*.

[27] Machavariani G., Lumer Y., Moshe I., Meir A., Jackel S. (2007). Efficient extra cavity generation of radially and azimuthally polarized beams. *Opt. Lett.*.

[28] Pohl D. (1972). Operation of a ruby laser in the purely transverse electric mode TE01. *Appl. Phys. Lett.*.

[29] Chen P., Ji W., Wei B., Hu W., Chigrinov V., Lu Y. Q. (2015). Generation of arbitrary vector beams with liquid crystal polarization converters and vector-photoaligned q-plates. *Appl. Phys. Lett.*.

[30] Shu W., Ling X., Fu X., Liu Y., Ke Y., Luo H. (2017). Polarization evolution of vector beams generated by q-plates. *Photonics Res.*.

[31] Arbabi A., Horie Y., Bagheri M., Faraon A. (2015). Dielectric metasurfaces for complete control of phase and polarization with subwavelength spatial resolution and high transmission. *Nat. Nanotechnol.*.

[32] Fan Q. (2020). Independent amplitude control of arbitrary orthogonal states of polarization via dielectric metasurfaces. *Phys. Rev. Lett.*.

[33] Fan Q. (2019). Broadband generation of photonic spin-controlled arbitrary accelerating light beams in the visible. *Nano Lett.*.

[34] Zheng G., Mühlenbernd H., Kenney M., Li G., Zentgraf T., Zhang S. (2015). Metasurface holograms reaching 80 % efficiency. *Nat. Nanotechnol.*.

[35] Huang L., Zhang S. S., Zentgraf T. (2018). Metasurface holography: from fundamentals to applications. *Nanophotonics*.

[36] Li X. (2022). Independent light field manipulation in diffraction orders of metasurface holography. *Laser Photonics Rev.*.

[37] Zhou H. (2020). Polarization-encrypted orbital angular momentum multiplexed metasurface holography. *ACS Nano*.

[38] Sande S., Bozhevolnyi S. I., Ding F. (2022). Broadband spin-multiplexed single-celled metasurface holograms: a comprehensive comparison between different strategies. *Nanophotonics*.

[39] Deng Z. (2018). Diatomic metasurface for vectorial holography. *Nano Lett.*.

[40] Deng Z. (2020). Full-Color complex-amplitude vectorial holograms based on multi-freedom metasurfaces. *Adv. Funct. Mater.*.

[41] Liu Y., Ling X., Yi X., Zhou X., Luo H., Wen S. (2014). Realization of polarization evolution on higher-order Poincaré sphere with metasurface. *Appl. Phys. Lett.*.

[42] Shu W. (2016). Propagation model for vector beams generated by metasurfaces. *Opt. Express*.

[43] Overvig A. C. (2019). Dielectric metasurfaces for complete and independent control of the optical amplitude and phase. *Light: Sci. Appl.*.

[44] Bernet S., Harm W., Marte M. R. (2008). Demonstration of focus-tunable diffractive Moire-lenses. *Opt. Lett.*.

[45] Bai X. (2022). Dynamic millimeter-wave OAM beam generation through programmable metasurface. *Nanophotonics*.

