# Supplementary material for: Cylindrical vector beam multiplexing holography employing spin-decoupled phase modulation metasurface
Source: Nanophotonics. 2024 Feb 7;13(4):529–38. doi: 10.1515/nanoph-2023-0731 (PMC11501858; doi:10.1515/nanoph-2023-0731)
Supplement: Supplementary file 1 — Supplementary Material Details [file j_nanoph-2023-0731_suppl_001.docx]

Supplementary Materials for
**Cylindrical vector beam multiplexing holography employing spin-decoupled phase modulation metasurface**

**Supplementary note S1:**


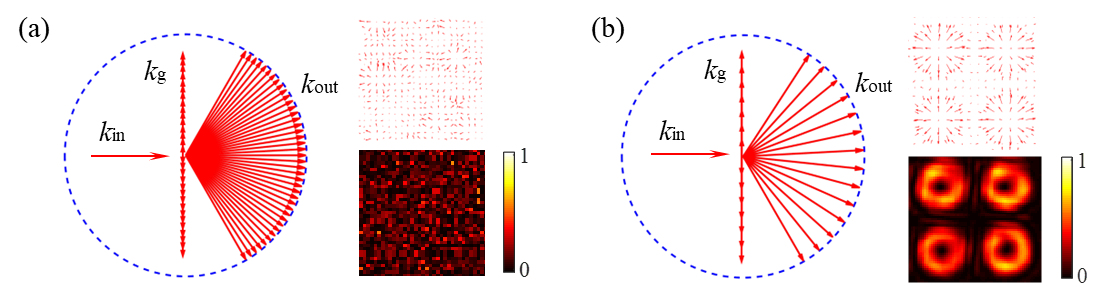


Figure S1 (a) Conventional digital hologram with a quasi-continuous spatial frequency distribution breaks the polarization distribution carried by an incident CVB; (b) CVB hologram design ideas which introducing discretization spatial frequency to preserve the polarization distribution of CVB;

Figure. S1 (a) shows the holography process with digital hologram, due to the quasi-continuous spatial frequency distribution of the conventional digital hologram, the light spot of the CVB is larger than the size of a single pixel, so that both polarization and light intensity distribution of the CVB will be destroyed because of the adjacent CVB overlap each other and interfere. To avoid spatial overlap of the polarization state of adjacent CVBs, it could add a linear spatial frequency shift to an incident beam (*k*_in_) to preserve the complete polarization distribution in each pixel of a reconstructed holographic image. In other words, the conventional digital hologram with a quasi-continuous spatial frequency should be discretized. As shown in Fig. S1 (b), the holographic pattern on the image plane is discretized by spatially sampling the holographic image, which increase the distance between adjacent pixels. Hence, it can avoid overlapping between CVBs in adjacent pixels by selecting a suitable sampling period, ultimately preserving the CVB modes in holographic images.

**Supplementary note S2:**

According to Fourier integral theorem, the light field distribution of imaging plane can be decomposed into an infinite number of plane-wave spatial frequency components as [1]:

$$E_{out}(x,y)=\iint E_{in}(k_{x},k_{y})\exp[-i(k_{x}x+k_{y}y)]dk_{x}dk_{y},$$

where (*x*, *y*) and (*k_x_*, *k_y_*) the represent the coordinates in the image plane and hologram plane, respectively. Hence, we can use the Fourier transform to connect the field distributions of two planes:

$$E_{I}(x,y)=fft(E_{H}(k_{x},k_{y})),$$

where *fft* denotes the fast Fourier transform. When we use the CVB to reconstruct the holographic image, the field of hologram plane can be expressed by:

,

where is the field of CVB, $(r,\theta)$ are cylindrical coordinates, $m$ is the polarization order of CVB. It should be noted that this z-component is weak and can be ignored under paraxial conditions. Theoretically, the CVB can be decomposed into two orthogonal circularly polarized sub-beams with orbital angular momentum (OAM). The Jones matrices can be expressed as:

$E_{vector}=\left[\begin{matrix} \text{cos(m}\theta) \\ \text{sin(m}\theta) \end{matrix}\right]=\frac{1}{2}\exp(-im\theta)\left[\begin{matrix} 1 \\ i \end{matrix}\right]+\exp\left(i\text{m}\theta\right)\left[\begin{matrix} 1 \\ -i \end{matrix}\right],$ (4)

Hence, to streamline the operation, we conducted a separate analysis of the two distinct components of CVB. For the left-handed component the field distribution of image plane can be obtained:

$E_{IL}=fft[E_{H}(x,y)\cdot\exp(im\theta)]=fft[E_{H}(x,y)]\otimes fft[\exp(im\theta)],$ (5)

where denotes the convolution operation. Similarly, it can be concluded that the field distribution of image plane with the left-handed component incident:

$E_{IR}=fft[E_{H}(x,y)\cdot\exp(-im\theta)]=fft[E_{H}(x,y)]\otimes fft[\exp(-im\theta)].$ (6)

In this case, we can obtain the field distribution of image plane when a CVB as the light source of holographic reconstruction:

(7)

From the Eq. (7) we can find that the field of image plane can be expressed by a convolution between the field of holographic image and the Fourier transform of a CVB. And the Fourier transform of a CVB acts as the kernel function of the convolution, can be copied in each pixel of the holographic image.

**Supplementary note S3:**

**
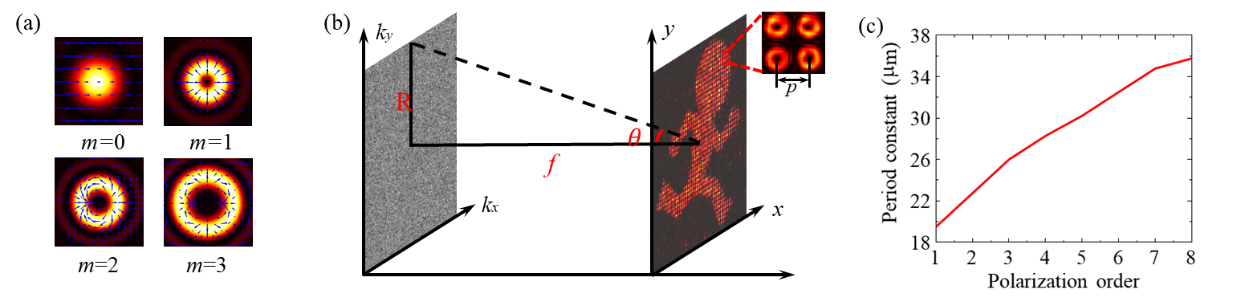
**

Figure S2 (a) Waist of CVB with different order; (b) the schematic illustration of reconstruction of CVB holography; (c) relationship curve of sampling spatial frequency (p) and polarization order (m) of CVBs.

Cylindrical vector beams (CVBs) are solutions to the wave equation in its vectorial form and obey axial symmetry in both amplitude and phase. The electric field of a CVB can be approximated as

$\vec{E}(\rho,\theta)=R(\rho)|m,\theta\rangle=R(\rho)\left[\begin{matrix} \sin(m\theta) \\ \cos(m\theta) \end{matrix}\right],$ (8)

where $(\rho,\theta)$ are cylindrical coordinates, $R(r)$ is a solution to the radial part of the wave equation, and $|m,\theta\rangle$ represent the spatially varying polarization state of CVB. Due to the cylindrical symmetry of CVB, the modulus amplitude of its spatial frequency spectrum can be calculated by [2]

$\left|E(r,\theta)\right|=abs\left|fft(E(\rho,\theta))\right|=\left|\frac{(-1)^{m+1}k}{f}\left[\begin{matrix} \sin(\mathrm{m}\theta) \\ \cos(\mathrm{m}\theta) \end{matrix}\right]\int_{0}^{R} J_{m}(\frac{k}{f}\mathrm{r}\rho)\rho d\rho\right|,$ (9)

which $k=2\pi/\lambda$is the wave number of incident beam, *f* is the focal length of a Fourier lens, R is the radius incident beam, r and ρ are the radius in the hologram plane and image plane, respectively. Due to the amplitude of CVB is radial symmetry, the amplitude is irrelevant to the azimuthal angle $\theta$, hence the Eq. (9) can be further simplification to:

$\left|E(r,0)\right|=\left|\frac{(-1)^{m+1}k}{f}\int_{0}^{R} J_{m}(\frac{k}{f}\mathrm{r}\rho)\rho d\rho\right|$ (10)

From Eq. (10), it can be found that the radius of CVB in the image plane was determined by the polarization order (m), the effective numerical aperture ($NA=\sin(\mathrm{atan}(R/f))$) and the wave number *k* of the incident beam, respectively. The light filed distribution of CVB with different polarization orders (m) has been shown in Fig. S2 (a), the radius of CVB is positively correlated with its polarization order. To avoid interference of CVBs between adjacent pixels at the image plane, the 2D Dirac comb function has been used to discretize the spatial frequency distribution of image. And the sampling constants p is dependent on the diameter of CVB at the image plane, where p should greater than or equal to diameter of CVB. In this work, when the wavelength is 633 nm and NA = 0.65, the relationship diagram between sampling spatial frequency (p) and polarization order (m) of CVBs has been shown in Fig. S2 (c).

**Supplementary note S4:**


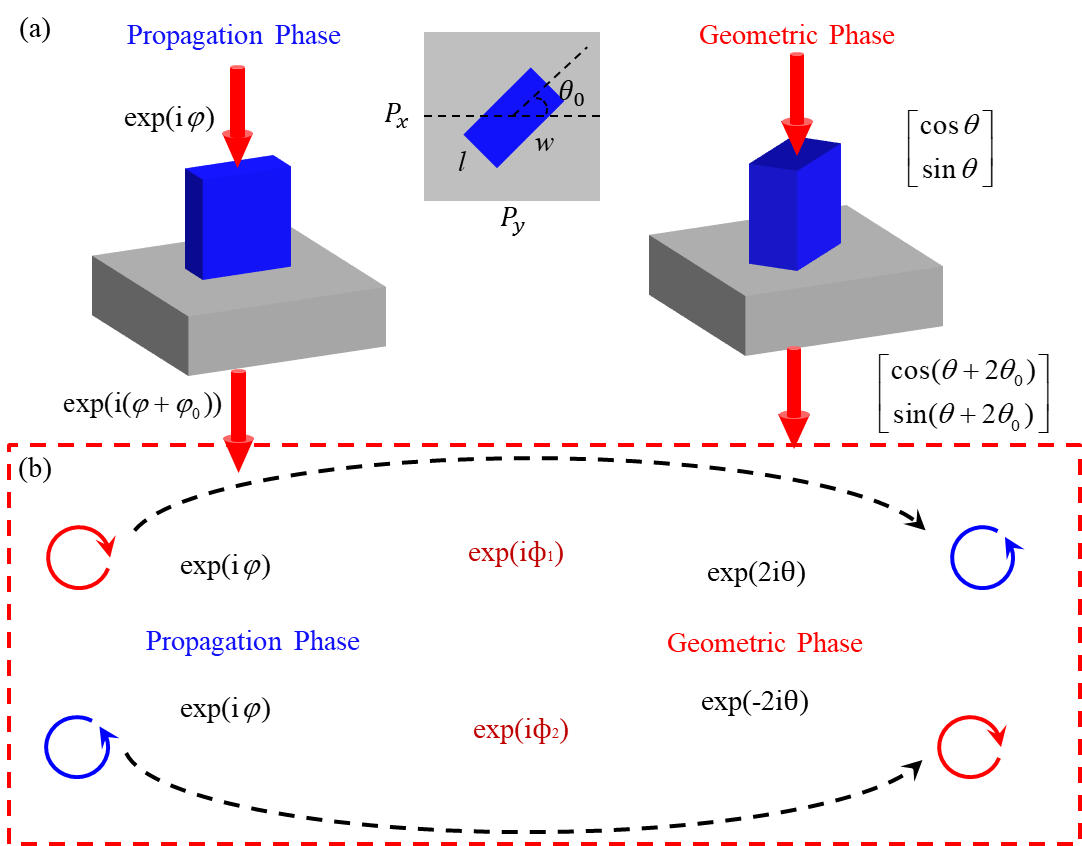


Figure S3 Schematic diagram of propagation and geometric phase metasurface.

Metasurfaces, subwavelength arrays of optical phase-shifting elements, provide an exciting platform for ultrathin optics. A distinguishing feature of metasurfaces is the sophistication with which the individual phase-shifting elements can be engineered. The two most common metasurfaces phase modulation types are propagation phase and Pancharatnam-Berry (P-B) phase. For the propagation phase metasurface, the working principle has been shown in Fig. S3 (a). When a beam of light passes through a metasurface composed of two media with different refractive indices, due to the boundary conditions are modified by the resonant excitation of an effective current within the metasurface, the reflection and propagation coefficients will be then dramatically changed. When the wavelength of incident light and the refractive index of the nano-antenna are fixed, the phase change mainly depends on the size of the nano-antenna and the polarization direction of the incident light. Here, we assume that the x- and y- direction polarization of incident light beam carry phase change $\psi_{x}$ and$\psi_{y}$, respectively. On the other hand, the P-B phase is a kind of polarization-dependent phase, in other words, the phase change stems from polarization change. If two parts of a uniformly polarized wave front are transported to a common polarization state along two different paths on the Poincaré sphere (polarization state space), a relative phase emerges between the two equals to half the solid angle ($\theta$) enclosed by the path. For example, the metasurface composed of half-wave plate elements ($\psi_{x}-\psi_{y}=\pi/2$), it can convert the RCP (LCP) to LCP (RCP) and imposes a phase $exp(2i\theta)$.

However, in the traditional phase modulation method by metasurfaces, neither the propagation phase metasurfaces nor the geometric (P-B) metasurfaces can independently modulate the phase of LCP and RCP beam. Here, we propose a phase modulation method that combines the P-B phase and the propagation phase, which can independent control the phase of LCP and RCP components of the incident beam. The principle diagram is shown in Fig. S2 (b), here we assume that the phase modulation for LCP and RCP are and , respectively. For the propagation phase, the phase difference between $\psi_{x}$ and $\psi_{y}$ is fixed, so we can only consider one of the components. Hence, we can get the following relationship:

(11)

(12)

where $exp(i\psi)$ is the propagation phase, and $exp(\pm2i\theta)$is the P-B phase. By solving the Eq. (11) and Eq. (12), we can get:

(13)

(14)

Hence, we can independently modulate the phase of LCP and RCP beam. In other words, after we determine the propagation phase and the geometric phase according to the phase transformations to be realized by the RCP and LCP, the size and rotation angle of the nano-antenna in each pixel can be further confirmed.

**Supplementary note S5:**


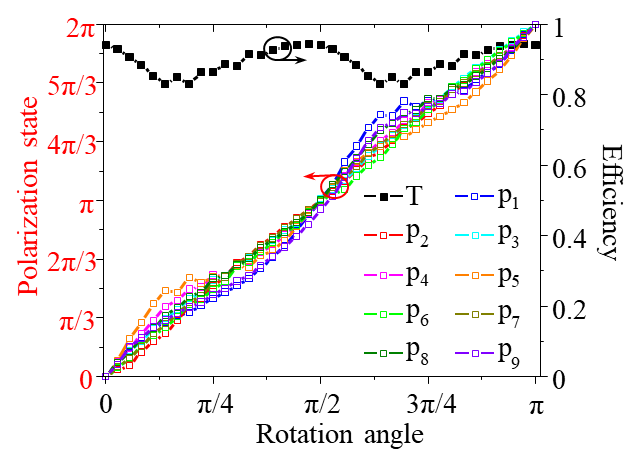


Figure S4 Numerical characterization of the polarization state and conversion efficiency with different azimuth angles of nanofin.

The curve of polarization state (linear polarization at different azimuth angles) and all nine of nanofin rotation angle for a x- direction linear polarization incident has been shown in Supplementary Fig. S4, the black curve represents the average polarization conversion efficiency. The simulation results are in good agreement with the theory (the polarized angle is twice of the included angle between incident polarized angle and azimuth of nanofin), the layout of the metasurface can further obtained base these simulated parameters.

**Supplementary note S6:**


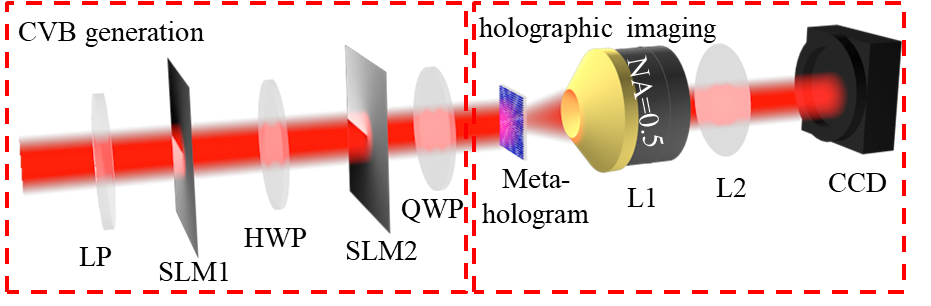


Figure S5. Experimental setup for the demonstration of metasurface based CVB holography.

The experimental setup consists of two parts: CVB generation and holographic imaging. In the experiment, an orthogonal polarization modulation method based on spatial light modulator has been used to generate incident CVB. The incident gaussian beam (633 nm) propagates through a linear polarizer and change to 45° polarized beam. Due to the spatial light modulator (SLM) can only modulate the wavefront of x-direction linearly polarized light. The SLM1 imprinted a helical wavefront with topological *l*=1 onto the x-polarized component of optical beam, and the half wave plate (HWP) exchanges the x and y polarized components in the beam, then the SLM2 imprinted a helical wavefront with topological *l*=-1 onto the y-polarized component of optical beam. Finally, a quarter wave plate (QWP) converts the x- (y-) polarization to left (right) circular polarization, which can obtain CVB with correspond polarization order. After that, the meta-hologram sample is placed in the focal plane of an objective lens (L1) (×40/NA = 0.65) to guarantee that the Fourier plane is located in the back focal plane. And the other lens (L2) is used for capturing the Fourier plane on a CCD camera, where the focal length of L2 is 50 mm.

**Supplementary note S7:**


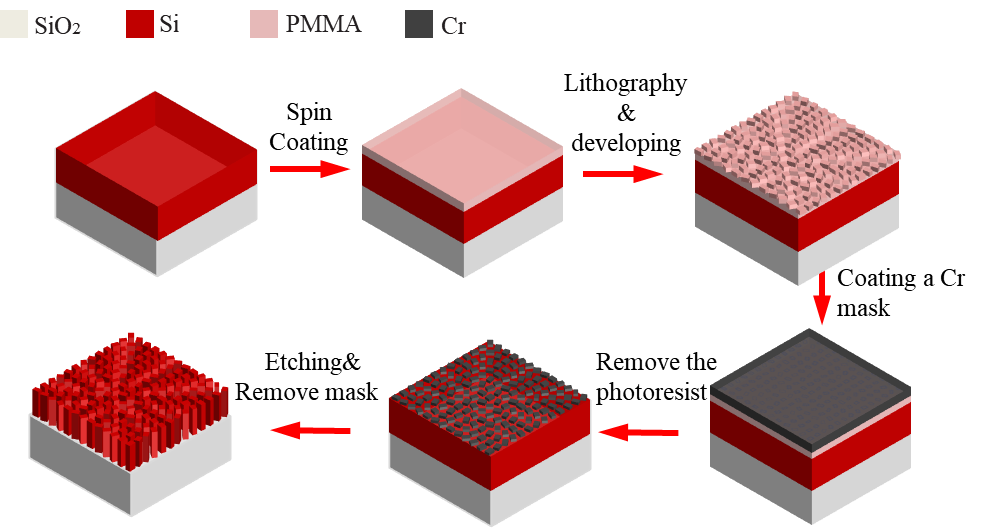


Figure S6 Preparation process of Si-SiO_2_ metasurface.

The Si-SiO_2_ based metasurface were fabricated through a standard Electron Beam Lithography (EBL) system (Raith BV, EBPG 5150). Frist of all, a layer of monocrystalline silicon (a-Si) film (thick of 400 nm) is depositing on a double-sided polished fused silica (SiO_2_) substrate by Magnetron sputtering. Then a 150nm thick photoresist (Polymethyl methacrylate, PMMA, 950K) is spin-coated on the prepared Si-SiO_2_ substrate by a homogenizer, which choosing a speed of 4000 rpm for 1 minute. And baking the resist at a temperature of 180 °C for 1 minute 30 seconds to solidify it. The desired structure was patterned by using EBL and the E-beam resist exposition was performed at 20 keV. After that, place the sample in 1:3 MIBK: IPA solution for 60 seconds to development, and washed with IPA for 30 seconds to fixation. Later, a 30nm thick chromium (Cr) layer is deposited on the sample by using the electron beam evaporator (ASB-EPI-C6), and the lift-off process is achieved in the 30% acetone solution for 6 hours. The desired patterns were transferred from Cr mask to Si by using the inductively coupled plasma reactive ion etching (ICP-RIE, NMC, GSE200Plus), which the plasma composed of SF_6_ and CHF_3_ mixed gases (SF_6_: CHF_3_= 1:7). Finally, the Cr mask on the top of Si nanoantennas was removed by using chemical etching with 1:2 solution of Ce(NH_4_)_2_(NO_3_)_6_ : HNO_3_.

**Supplementary note S8:**

In this simulation, all results were performed by using the Lumerical FDTD Solution (a commercial software). The x- and y- polarized light beam with wavelength of 632 nm (propagate along z-direction) are normally incident onto a single nanopillars, while the periodic boundary condition was used along x- and y- directions, and the perfect matched layer (PML) was chosen along z-direction. According to the Nyquist sampling criterion, here we set the period of meta-atoms as 300 nm and height is 400 nm. The refractivity of Si in this simulation is set to 3.88, SiO_2_ is 1.46. After sweeping the geometric parameters of the nanopillar (including the size and thickness), the transmission efficiency and phase delay and of *x*- and *y*-direction can be obtained, respectively. In order to cater to the actual processing conditions, here we set the scanning parameters of length and width from 50 nm to 250 nm. On the premise of meeting the half-wave condition (, , are the phase delay of *x*- and *y*-direction, respectively), we find 9 level propagation phases uniformly distributed between 0 to 2π. For the simulation of metasurface, the number N of unit cell in a transverse plane is set as 60×60, and the transversely simulated area is set as 18×18$\mu m^{2}$.

**Supplementary note S9:**


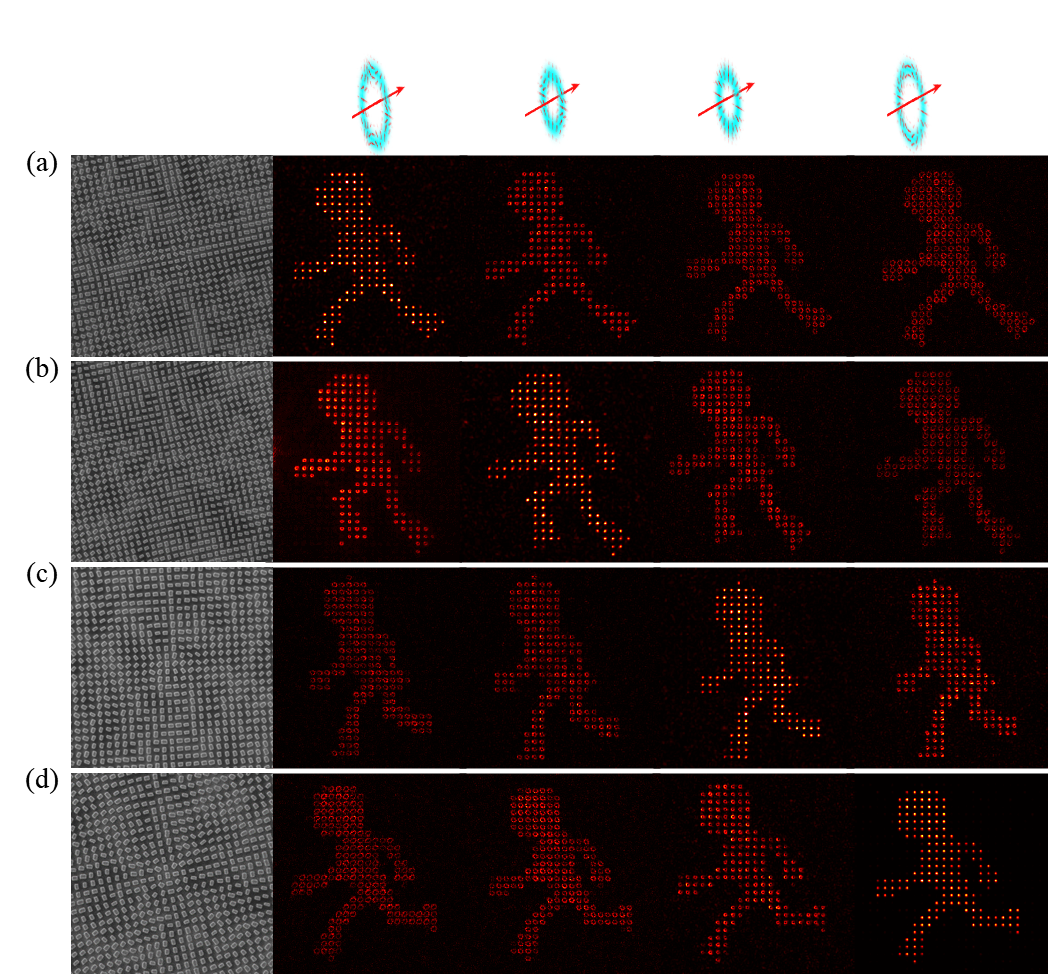


Figure S7 Experimental characterization of CVB-selective meta-holograms based on the illumination of the four CVBs with m=−2, −1, 1, 2.


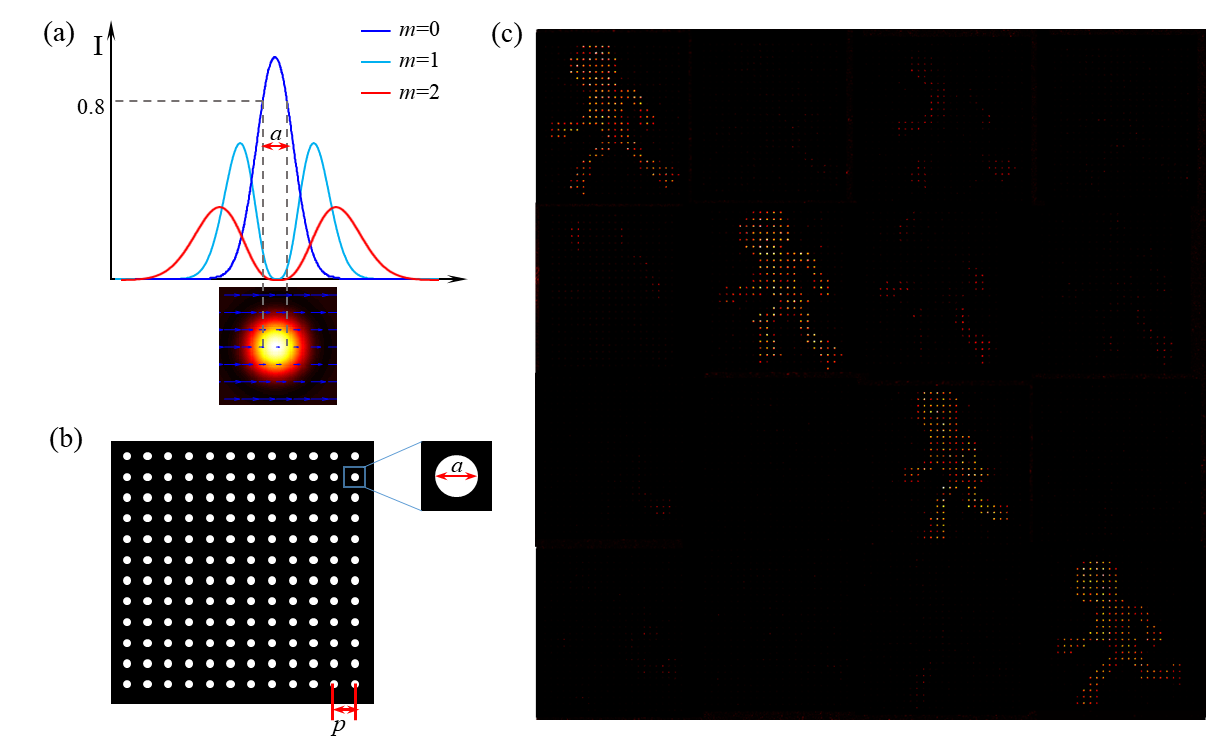


Figure S8 (a) The intensity cross-sections of CVB with m=0, 1, 2; (b) Schematic of a filtering aperture array, where p is the periodicity, a is the diameter of aperture. (c) Light intensity distribution of each CVB channel after through the aperture.

As show in Fig. S7, when the CVB with different polarization orders from -2 to 2 illuminating to the meta-hologram, the shapes of reconstructed holographic images remain unchanged, but the polarization order of CVB mode in each pixel is related to the incident light. At the holographic image plane, according to the conservation of CVB mode, the CVB mode with an inverse polarization order (-m) can recover the fundamental gaussian mode in each pixel of the holographic image. The light beam in each pixels of images on other channels are converted to CVBs with non-zero polarization order. In this case, as show in Fig. S8(a), the gaussian beam has relatively stronger intensity distribution and smaller diameter compared to the CVB with non-zero order. Hence, the selective imaging can be realized by using an aperture array to filter the non-zero order CVB. Figure S8(b) shows the diagram of sample aperture, it composed of arrayed circular holes with a diameter of a. There are two key parameter of the sample aperture that are period constants (*p*) and diameter of sample aperture size *a*. The *p* was determined by the polarization order, the effective numerical aperture and the wave number of the incident beam, and the relationship curve between *p* and polarization order of CVBs has been shown in Fig. S2(b). The sample aperture size *a* is depending on the diameter of the recovered Gaussian spot, it influenced crosstalk between adjacent channels. To reduce crosstalk, here we set the sample aperture size equal to the spot diameter at a light intensity of 0.8. Figure S8(c) shows the light intensity distribution of each CVB channel after through the aperture, it can be seen that only the corresponding holographic image can be imaged through the aperture. It also proves that CVB holography has the characteristic of selective imaging.

**Supplementary note S10:**

To analysis the crosstalk between different channels in the CVB multiplexing holography, here we calculated the signal-to-noise ratio (SNR) of each CVB holography channels. The SNR can be defined as $SNR=10\times\log(\frac{I_{Signal}}{I_{Noise}})$, where I*_Signal_* and I*_Noise_* represent the integration intensity of holographic images reconstructed from the desired CVB information channel and other CVB multiplexing channels, respectively. In this case, as show in Fig. S9(a), the SNR of four channels in the experiment are equal to 11.5dB, 14.8dB, 11.3dB and 9.16dB, respectively.

When the crosstalk increases to a specific value, it is impossible to extract holographic image information from the multiplexing information. Hence, there must be a maximum value of multiplexing channels. In this case, we set the mode intervals as 1 and simulated the multiplexing of multiple CVB modes, the relationship between the numbers of multiplexing and crosstalk has been show in Fig. S9(b). Here, we assume that the image can only be demultiplexed when the SNR is greater than 3dB. Hence, in this work, the theoretical limit number of CVB could be multiplexed is 78 in an ideal environment. In fact, the crosstalk is related to the numbers of multiplexing, mode intervals between adjacent channels, aperture of filter, type of holography and even the optimization algorithms of hologram generation.


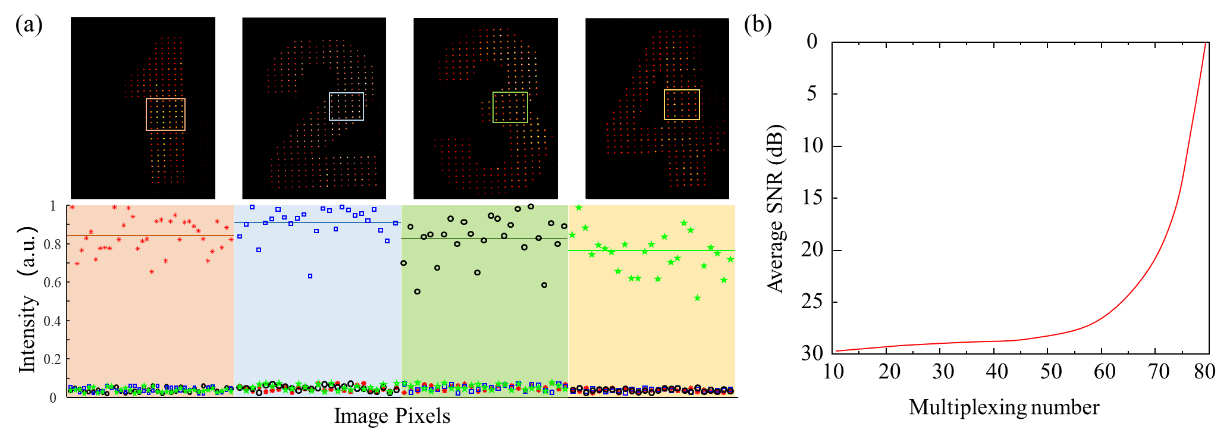


Figure S9 (a) SNR of each channels; (b) Relationship curve between the multiplexing numbers and crosstalk in the simulation.

To reduce crosstalk between adjacent channels, in the other words, to increase SNR of each channels. There are several methods, firstly, increase the mode interval between adjacent channels. As show in Fig. S2(a), the CVB is a hollow beam with “donut” structure, it can be seen that the hollow diameter is increased with the polarization order increases. In this case, with the increased of mode interval, after reconstruction of the target image, the crosstalk between CVB of adjacent channel and recovered gaussian beam is reduced.

In addition, another method to influence the crosstalk between adjacent channels is that change the size of filter aperture. Figure S8(a) shows the cross-section distribution of CVB light intensity, to separate the fundamental gaussian mode from CVB with non-zero order, an aperture array with diameter of a can be used filter out the gaussian mode to achieve the demultiplexing of target image. Hence, in theory, the smaller the diameter of aperture (a), the smaller the crosstalk between channels, but the lower the imaging light intensity. In this case, it is necessary to choose the appropriate diameter according to the actual situation.

Finally, we modulated the phase and polarization of the light to realize the CVB mode holography in this work. Such a holographic imaging method is a phase only method to reconstruct the target image. Hence, if we can modulate the complex-amplitude of incident light beam to achieve the amplitude-phase mixed holography, it can realize exact convolution between a complex-amplitude image channel and the CVB wave-front to reduce the crosstalk between channels.

**Supplementary note S11:**

*
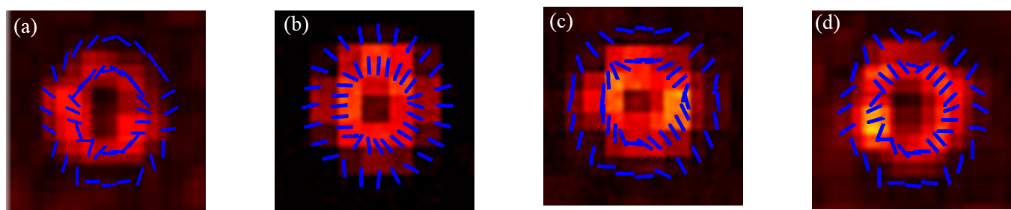
*

Figure S10 Polarization distribution of CVB in each pixels of reconstructing holographic images at the imaging plane.

**Reference**

1. H. Ren, G. Brierer, X. Fang, et al., Metasurface orbital angular momentum holography, Nat. Commun., vol. 10, no. 1, p. 2986, 2019.
2. V. Kotlyar, S. Khonina, A. Kovalev, et al., Diffraction of a plane, finite-radius wave by a spiral phase plate, Opt. Lett., vol. 31, no. 11, pp. 1597–1599, 2006.
